# Simulation of an electrically actuated cantilever as a novel biosensor

**DOI:** 10.1038/s41598-020-60296-9

**Published:** 2020-02-25

**Authors:** Masoud SoltanRezaee, Mahdi Bodaghi

**Affiliations:** 1 Department of Mechanical Engineering, Tarbiat Modares University, Tehran, Iran; 2 Department of Engineering, School of Science and Technology, Nottingham Trent University, Nottingham, NG11 8NS United Kingdom

**Keywords:** Biosensors, Mechanical engineering, Biosensors

## Abstract

Recently, detecting biological particles by analyzing their mechanical properties has attracted increasing attention. To detect and identify different bioparticles and estimate their dimensions, a mechanical nanosensor is introduced in this paper. To attract particles, numerous parts of the substrate are coated with different chemicals as probe detectors or receptors. The principal of cell recognition in this sensor is based on applying an electrical excitation and measuring the maximum deflection of the actuated cantilever electrode. Investigating the critical voltage that causes pull-in instability is also important in such highly-sensitive detectors. The governing equation of motion is derived from Hamilton’s principle. A Galerkin approximation is applied to discretize the nonlinear equation, which is solved numerically. Accuracy of the proposed model is validated by comparison studies with available experimental and theoretical data. The coupled effects of geometrical and mechanical properties are included in this model and studied in detail. Moreover, system identification is carried out to distinguish bioparticles by a stability analysis. Due to the absence of a similar concept and device, this research is expected to advance the state-of-the-art biosystems in identifying particles.

## Introduction

Biosensors have been introduced to detect different characteristics of biological particles. They have numerous applications in biotechnology as biomass detectors^1,2^, thermal switches^3–6^, smart resonators^7–12^, and other biomedical devices^13–24^. Due to the important biological applications of such systems in the drug delivery, clinical diagnostics, measurement, and organism detection, many scientists have studied their features such as operational range, instability, and performance^8,24,25^. In recent decades, theoretical investigations of different micro and nanoscale systems have attracted much attention^26–28^, which can extremely be useful in this multidisciplinary field. Recently, developments in precision engineering have enabled the fabrication of advanced miniature instruments like micro and nanoelectromechanical system (MEMS and NEMS)^23,29,30^. Moreover, experimental advances in biotechnology have demonstrated a considerable ability to recognize cells within these precise bioM/NEMS^19,31^.

With high demands for ultra-sensitive biosensors, beam-based micro and nanoresonators have emerged and developed due to their outstanding electrical and mechanical characteristics. Studying the pull-in instability, which can restrict the operational range of micro and nanosystems is essential in the modeling and analysis of biosensors. Almost all the types of biosensors consist of a suspended conductor and a fixed plate. The adsorption of the target biomolecules on any of electrodes changes its stiffness and displacement. It is demonstrated that the sensitivity of a sensor is maximized close to its critical point (pull-in characteristics), where the effective stiffness vanishes. Moreover, it was illustrated that the sensitivity of the system in measuring the adherent cell mass can be increased by decreasing deformable electrode dimensions^32^.

It was experimentally demonstrated that cantilever electrodes have a dramatic potential to be used as mechanical biosensors^9,10,33–35^. The stiffness of cantilever beams is lower than clamped-clamped ones. Therefore, their sensitivity is considerable, which is very important in biosensing applications. The ability of microcantilever arrays for selective immobilization and fast quantitative recognition of biological entities has been illustrated experimentally^34,35^. Moreover, smart piezoelectric materials have widely been used to examine mechanical behaviors of microdetectors^7–12^ and atomic force microscope probes^7^. A novel platform has been introduced to apply as an ultra-sensitive microsensor based on cantilevers patterned with crosslinked hydrogels^36^. Gupta *et al*.^37^ studied the detection of bacterial cells and antibodies by employing surface machined especial manipulators. Wee *et al*.^9^ experimentally examined the electrical detection of disease markers employing piezoresistive microcantilevers due to antigen–antibody interaction. The results illustrated that the interaction produces a compressive tension on the microelectrode, which results in beam bending and resistance change of the surface layer. Microcantilevers excited by the piezoelectric layer as both sensors and actuators have been developed to detect biomaterials like proteins and DNA^10,12^. Afterward, microfabricated detectors based on the mechanical motions have been studied to be used as polymer biochemical sensing tools^11^. Chen *et al*.^38^ studied a rapid label-free detection of disease-related proteins employing microelectrodes. The results demonstrated that the sensitivity depends on the electrode material and geometry. Biosensing behaviors of micro and nanocantilevers have been reviewed with considering the nanobiotechnology^33^, pathogens rapid recognition^24^, microfabrication^39^, tool platform^31^, dynamic-mode^40^, biomedical applications^22^, mechanical applications^41^, and disease detection^20^. Furthermore, biological purposes have been summarized with attention to biorecognition fields, surface functionalizations, and resonant frequency^39,40,42^. The results of this review indicate the important role of cantilevers in biosensors as a key component.

Mehdipour *et al*.^1^ developed a novel biomass detector for simulating vibrations of a cantilevered single-walled carbon nanotube. The sensitivity of the biosensor to the values and positions of the attached mass was calculated with consideration of the pull-in instability. An actuator was examined by Shaat and Abdelkefi^16^ for mass detection of biocells and materials characterization. Furthermore, for disease diagnosis aims, a micromechanical actuator was suggested to detect human immune-viruses (HIVs). Later, they^2^ developed a nanocrystalline silicon antibody-coated cantilever to investigate the pull-in instability and sensitivity of biocell nanosensors using modified couple stress theory (MCST). However, they modeled the effects of surface layer energy and material size, neglecting the fringing field and dispersion forces (Casimir and van der Waals (vdW)) for more simplicity. The lateral vibrations of rectangular microplates with applications in industrial or biological detectors were modeled to identify the mass and its position by obtaining eigenvalues and resonant frequencies^26^. Overall, investigating instability characteristics and frequency analysis are significant in the design of actuated biological nanosystems.

The adsorption of target particles on either the suspended electrode or the fixed one changes the mechanical behavior of systems. For detection purposes, two different scenarios can be considered in beam-based bioM/NEMS. In the first case, the surface of the deformable electrode is coated with chemical detectors, probe molecules, or antibodies, which can attract analytes, molecules, or other target particles^37^. Several systems based on this classical scenario have been designed and analyzed in detail, especially as biomass detectors^1,2,16,26,43^. In this condition, the main methodology is that by measuring the variation of resonance frequencies, we will be able to estimate the attracted mass. There is another scenario, particularly to distinguish the size of bioparicles. In this case, which has less been considered, the fixed conductor is coated with materials that can capture target components. Hence, the component will block a part of the actuated substrate leading to a decrease in the effective length of the sensing piece. This phenomenon not only affects the instability voltage, but also the electrode deflection. Consequently, we will be able to measure the biological particles by investigating the pull-in instability characteristics.

The main objective of this research is to employ actuated beam-based systems as practical biosensors, which can also be applied as material detectors. In order to enable simulation and analysis of such biosensors, a nanomanipulator is developed. To maximize the sensitivity of the detector, a cantilever nanobeam is considered as a deformable electrode. Numerous parts of the substrate are coated with different chemicals as receptors to attract biological components. By applying the electric potential and analyzing pull-in characteristics, we can recognize adherent components and their size. This model contains contributions of mechanical properties of actuated nanostructures, including the material length-scale, surface layer energy, nonlinear curvature, and dispersion effects. Moreover, the effects of electrode dimensions, i.e. thickness, width, length, and initial gap on the performance of biological devices are investigated. A frequency analysis of vibrating NEMS is implemented to examine system resonances. Owing to the absence of such concept and device in the open literature, this research would advance the state-of-the-art biosensors simulation with the capability of identifying biological particles.

Identifying the substance of particles in addition to their dimensions is not achievable by classical methods such as the resonance frequency analysis. Therefore, obtaining a practical approach to this end is desirable. Detecting biological component becomes possible by means of the introduced sensor. In addition, estimating dimensions of different particles is another capability of this device that is essential in biomedical researches. We will be able to determine the particle dimension by investigating the pull-in voltage and deflection of the electrode tip. Furthermore, the particle or target can be any suspended or segregated bioparticle that we want to detect and determine its dimensions by attracting it to the biodevice substrate. The particle size is vital because incubation can depend on it^44^. This instrument could have several biomedical applications, for example, it could be utilized in a variety of gas or liquid samples such as blood samples. Moreover, due to the ultra-small size of the current sensor, injection of a developed biocompatible one into the animal body would be accessible.

## Modeling and Methodology

**Figure 1:**
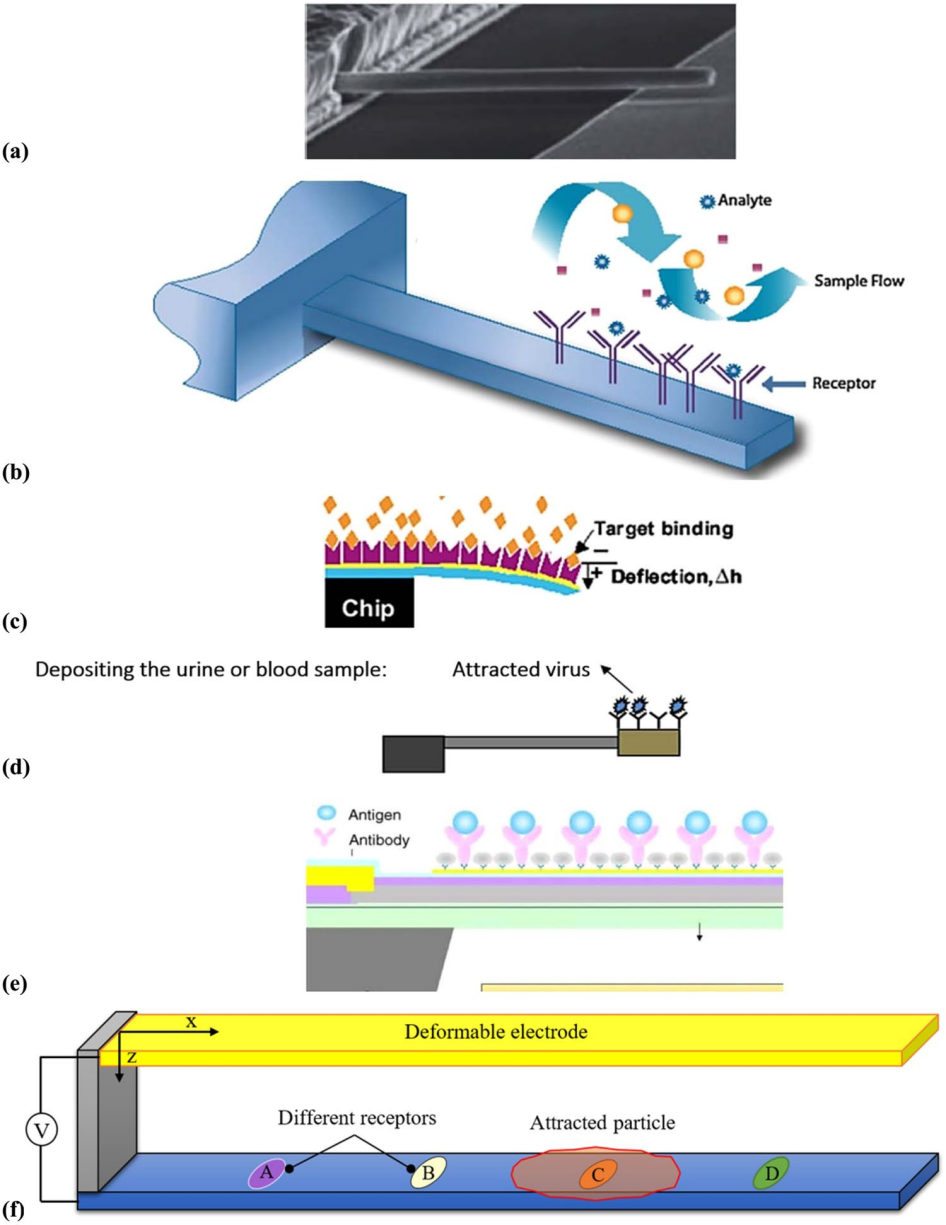
(**a**) An SEM image of a cantilever microbeam^45^, (**b**) a sensor in a liquid sample with receptors to attract analytes^40^, (**c**) a cantilever beam molecular detection (bending deflection changes due to binding of free antigen on the functionalized surface by antibodies)^30^, (**d**) an antibody-coated tip-mass beam in the blood sample^16^, (**e**) interaction of antigens with antibodies on different locations of the deformed vibrating electrode^9^, (**f**) a schematic of our proposed biosensor with an ability to detect different biological components (a biological particle adheres to one of the coated receptors).

Figure 1(a) shows an SEM image of a cantilever microbeam composed of a deformable electrode suspended over the substrate as the fixed conductor^45^. In Fig. 1(b), a schematic of a biosensor that exists in a liquid sample is presented^40^, where the deformable electrode has coated with a chemical that attracts especial analytes. Figure 1(c) displays a schematic of another cantilever biodetector, where the specific molecular binding on the surface induces a measurable bending deflection originating from the mechanical properties change^30^. Figure 1(d) demonstrates a typical schematic of an antibody-coated tip-mass beam in a blood sample, which can detect adherent entities by evaluating the change in the resonance frequency^16^. Figure 1(e) also displays a schematic of a microfabricated cantilever, which can attract antigens with coated antibodies on the external surface^9^. The movable arm deflects toward the fixed conductor gradually due to the applied potential difference and deposited mass. Finally, an NEMS biosensor is proposed in this work, see Fig. 1(f). It can identify different particles and their size by considering the critical applied voltage and maximum deflection of the electrode.

In the following, the nonlinear equation of motion (EOM) of the present miniature sensor is derived, by accounting effects of length-scale and surface layer in addition to electrostatic and Casimir attractions with consideration of the nonlinear curvature. The length, width, and height of the suspended electrode are denoted by *L*, *b*, and *h*, respectively. The initial gap and electric potential difference between the substrate and the electrode are *G* and *V*, respectively. The mechanical properties of the bulk material are Young’s modulus *E*, Poisson’s ratio *v*, the moment of inertia *I*, and beam density *ρ*.

Experimental results demonstrated that the mechanical characteristics of ultra-small systems could be different. The emergence of several substantial influences within the dimensional variation causes various new behaviors that are emerging in micro and nanoscales, which are not generally effective in other dimensions.

According to the modified couple stress theory^46^, which is a well-known nonclassical continuum elasticity theory, the strain energy arising from considering the size effect for micro and nanostructures can be expressed as^46^

1 $${U}_{l}=\frac{1}{2}(\frac{E}{2(1+\nu )})(bh){l}^{2}{\int }_{0}^{L}{(\frac{{\partial }^{2}w(x,t)}{{\partial }^{2}x})}^{2}{\rm{d}}x,$$

where *l* and *w*(*x, t*) indicate the small length-scale parameter and the beam transverse displacement (along the *x*-axis), respectively.

In the nanoscale, it is assumed that a surface layer, which encompasses around the bulk material, has different properties. Due to the considerable ratio of surface area to volume of oelectrodes, surface layer effects become more important for nanostructures. The terms *E*_s_ and *τ*_s_ indicate the elastic modulus and residual stress of the electrode surface layer, respectively. Furthermore, *EI* denotes the beam flexural rigidity. For a beam by considering the surface layer energy, one can obtain^47^

2 $${E}_{eff}{I}_{eff}=EI+{E}_{s}{I}_{s}=E\frac{b{h}^{3}}{12}+{E}_{s}(\frac{b{h}^{2}}{2}+\frac{{h}^{3}}{6}).$$

Moreover, the effective transverse distributed force due to the residual surface tension at the surface is

3 $$q(x,t)=2{\tau }_{s}b\zeta (x,t),$$

where *ζ* is the nonlinear curvature of the beam-based electrode. Consequently, the associated strain energy arising from this load is given by^47^

4 $${U}_{s}=\frac{1}{2}2{\tau }_{s}b{\int }_{0}^{L}\zeta (x,t)w(x,t)\,{\rm{d}}x.$$

When an external electric difference applies across two conductors, the conductor will undergo a relatively considerable deflection. As a result, the nonlinear curvature effect will become important for cantilever beams, where the strain at the neutral axis will remain zero. Therefore, the nonlinear curvature can be expressed as^48^

5 $$\zeta (x,t)=\frac{{\partial }^{2}w(x,t)}{\partial {x}^{2}}+\frac{1}{2}{(\frac{\partial w(x,t)}{\partial x})}^{2}\frac{{\partial }^{2}w(x,t)}{\partial {x}^{2}}.$$

Finally, the strain energy of the nanoelectrode subjected to large deformations with consideration of the around layer effects is written as^48^

6 $${U}_{m}=\frac{1}{2}{E}_{eff}{I}_{eff}{\int }_{0}^{L}{\zeta }^{2}(x,t)\,{\rm{d}}x,$$

The electrical attraction by considering the fringing field correction is given by^49^

7 $${F}_{els}=\frac{{\varepsilon }_{0}b{V}^{2}}{2{(G-w(x,t))}^{2}}(1+0.65\frac{G-w(x,t)}{b}),$$

where *ε*_0_ = 8.85 × 10^−12^F/m is the permittivity factor of vacuum.

The Casimir force is another significant effect for small-scale structures that is negligible in macroscales. This attraction has fundamental impacts on behaviors and responses of micro and nanosystems, which can be expressed as^50^

8 $${F}_{cas}=\frac{{h}_{P}b{\pi }^{2}c}{240{(G-w(x,t))}^{4}},$$

where *h*_*P*_ = 2π × 1.05457 × 10^−34^ J.s is Planck’s constant and *c* = 2.998 × 10^8^ m/s is the light speed.

Generally, to detect biological components, the fixed conductor is able to attract different particles owing to different chemicals that coat its surface. Therefore, the system is partly actuated, where the adherent particle blocks the substrate surface. As a result, the whole area of the fixed conductor cannot pull the deformable electrode and there is just a piecewise actuation in the biosensor. In this condition, the effective actuated area can be determined using the Heaviside function as follows

9 $$HS(x)=1-H(x-\underline{a})+H(x+\underline{b}-L),$$

where *a* and *b* are the length of active pieces and the part between these pieces has been blocked due to the attracted particle. It should be noted that the influence of biomaterials in changing the gap dielectric could be considered to improve the core model in the future development efforts. In this research, since the focus is to investigate certain effects, we assume that the sensor is calibrated before operating.

Since the biosystem is subjected to both electrical and molecule interaction forces, the performed work with consideration of piecewise actuation due to the adherent mass can be derived as

10 $${W}_{ext}={\int }_{0}^{L}({\int }_{0}^{w}({F}_{els}+{F}_{cas})HS(x)\,{\rm{d}}w(x,t))\,{\rm{d}}x.$$

Moreover, the kinetic energy of the electrode is given by

11 $${E}_{k}=\frac{1}{2}{\int }_{0}^{L}\rho (bh){(\frac{\partial w(x,t)}{\partial t})}^{2}{\rm{d}}x.$$

To establish EOM, a Hamiltonian approach is applied as

12 $$\delta {\int }_{0}^{t}({U}_{l}+{U}_{s}+{U}_{m}-{W}_{ext}-{E}_{k})dt=0.$$

Inserting kinetic and strain energies as well as the work performed by electrical and molecular forces into Eq. (12), the governing EOM of an actuated biosensor subjected to the cells by accounting size-dependency and surface layer under nonlinear curvature is derived as

13 $$\begin{array}{c}\frac{bhE{l}^{2}}{2(1+\nu )}\frac{{\partial }^{4}w}{\partial {x}^{4}}+(E\frac{b{h}^{3}}{12}+{E}_{s}(\frac{b{h}^{2}}{2}+\frac{{h}^{3}}{6}))[\frac{{\partial }^{4}w}{\partial {x}^{4}}+\frac{\partial }{\partial x}(\frac{\partial w}{\partial x}\frac{\partial }{\partial x}(\frac{{\partial }^{2}w}{\partial {x}^{2}}\frac{\partial w}{\partial x}))]\\ -b{\tau }_{0}\frac{{\partial }^{2}w}{\partial {x}^{2}}(4+{(\frac{\partial w}{\partial x})}^{2})+\rho bh\frac{{\partial }^{2}w}{\partial {t}^{2}}\\ =(\frac{{\varepsilon }_{0}b{V}^{2}}{2{(G-w)}^{2}}(1+0.65\frac{G-w}{b})+\frac{{h}_{P}b{\pi }^{2}c}{240{(G-w)}^{4}})(1-H(x-\underline{a})+H(x+\underline{b}-L)).\end{array}$$

The nonlinear EOM is rewritten in a non-dimensional form, where the subsequent model parameters are arisen:

14 $$\begin{array}{c}X=\frac{x}{L},\,W=\frac{w}{G},\,\alpha =\frac{\underline{a}}{L},\,\beta =\frac{\underline{b}}{L},\,HS(X)=1-H(X-\alpha )+H(X+\beta -1),\\ \phi =0.65\frac{G}{b},\,\xi =\frac{{G}^{2}}{{L}^{2}},\,\iota =\frac{6{l}^{2}}{(1+\nu ){h}^{2}},\,\eta =\frac{2{E}^{s}}{E}(\frac{3}{h}+\frac{1}{b}),\,\lambda =\frac{24{\tau }_{0}{L}^{2}}{E{h}^{3}},\\ {c}_{cas}=\frac{{h}_{P}b{\pi }^{2}c{L}^{4}}{240EI{G}^{5}},\,T=\frac{ht}{2{L}^{2}}\sqrt{\frac{E}{3\rho }},\,\upsilon =\frac{V{L}^{2}}{hG}\sqrt{\frac{6{\varepsilon }_{0}}{hGE}}.\end{array}$$

*X*: dimensionless length according to beam length,

*W*: dimensionless beam midpoint displacement,

*α*: dimensionless active length from electrode base to particle edge,

*β*: dimensionless active length from electrode tip to particle edge,

*HS*: Heaviside step function,

*ϕ*: ratio of initial gap to beam width,

*ξ*: square ratio of initial gap to beam length,

*ι*: material size dimensionless parameter,

*η*: surface elasticity dimensionless parameter,

*λ*: residual surface stress dimensionless parameter,

*c*_*cas*_: Casimir dimensionless coefficient,

*T*: dimensionless time,

*υ*: dimensionless voltage.

After inserting the above-mentioned terms into Eq. (13), and doing a few transformations, the general EOM can be derived as the following non-dimensional form. Moreover, boundary conditions (BCs) of the cantilever beam can be found in the open literature. It should be noted that by ignoring the last term, the static equation of equilibrium will be obtained.

15 $$\begin{array}{c}(\frac{{\upsilon }^{2}}{{(1-W)}^{2}}(1+\phi (1-W))+\frac{{c}_{cas}}{{(1-W)}^{4}})HS(X)=(1+\eta +\iota )\frac{{\partial }^{4}W}{\partial {X}^{4}}-2\lambda \frac{{\partial }^{2}W}{\partial {X}^{2}}\\ \,+\,\xi ((1+\eta )[\frac{\partial }{\partial X}(\frac{\partial W}{\partial X}\frac{\partial }{\partial X}(\frac{{\partial }^{2}W}{\partial {X}^{2}}\frac{\partial W}{\partial X}))]-\frac{1}{2}\lambda {(\frac{\partial W}{\partial X})}^{2}\frac{{\partial }^{2}W}{\partial {X}^{2}})+\frac{{\partial }^{2}W}{\partial {T}^{2}}.\end{array}$$

It should be noted that the weight force is ignorable in comparison with other forces^51–55^. A Galerkin approximation is then applied to reorganize the partial differential equation as a set of ordinary differential equations by introducing appropriate basic functions. Therefore, the vertical displacement of the electrode *W* is defined as a linear combination of independent modes as

16 $$W=\mathop{\sum }\limits_{i=1}^{N}{B}_{i}{\varphi }_{i}(X)={{\bf{B}}}^{T}{\boldsymbol{\varphi }},$$

where *B* is the amplitude parameter and *φ*_i_ are dimensionless eigenfunctions of cantilevers defined as

17 $${\varphi }_{i}=(\sin ({\varUpsilon }_{i}X)-\,\sinh ({\varUpsilon }_{i}X))\frac{\cosh \,{\varUpsilon }_{i}-\,\cos \,{\varUpsilon }_{i}}{\sinh \,{\varUpsilon }_{i}-\,\sin \,{\varUpsilon }_{i}}+\,\cosh ({\varUpsilon }_{i}X)-\,\cos ({\varUpsilon }_{i}X)$$

in which the values of ϒ_*i*_ will be calculated by considering the transcendental relation of beams with clamped-free BCs.

In instability circumstances, the tangent stiffness of structures must be singular^47^. As a result, there is an instrumental way to determine instability parameters (both pull-in voltage and deflection) of manipulators. For numerically solving the nonlinear differential equation of motion, the SSLM technique^56^ will be implemented. Note that the term *W*^*i*^ is the dimensionless deflection due to the external voltage *υ*^*i*^. By increasing the external excitation *υ*^*i*+1^ → *υ*^*i*^ + *δυ*, the electrode tip deflection is computed as *W*^*i*+1^ → *W*^*i*^ + *δW*.

Moreover, the resonance frequency must be zero, when a mechanical vibrating system collapses. In other words, by solving the nonlinear eigenvalue problem and looking for its non-trivial solutions, the responses of the dynamic problem will be calculated. Consequently, it is possible to obtain the resonance frequency by considering a process according to the dynamic governing equations. Determining the dynamic critical potential difference of electrically actuated sensors is also achievable.

## Parametric Study

Having the developed mathematical model of the electrically actuated ultra-small system, a parametric study of the present biosensor is conducted to analyze the effects of different parameters. Afterward, we will be able to identify and detect biological particles by investigating instability characteristics quantitatively and qualitatively.

**Figure 2:**
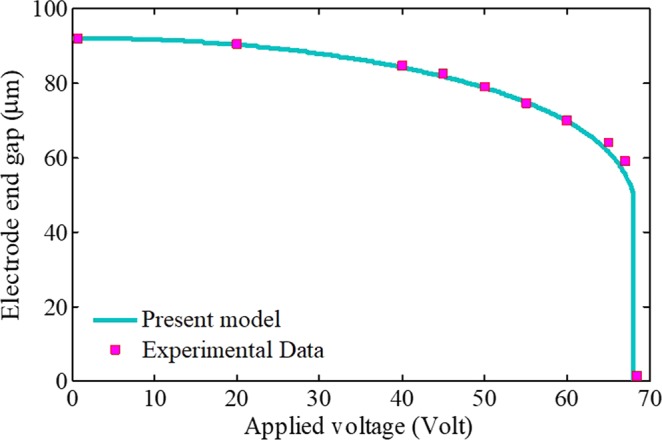
Comparison of the electrode tip gap calculated by the proposed model with experiments^57^.

Firstly, the system model and obtained results are verified with theoretical (Table 1) and experimental (Fig. 2) results. To this aim, a cantilever beam in the presence of the electrostatic attraction is considered and the pull-in voltage of the structure is obtained using the present model and the SSLM method. Afterward, it is compared with available results^51–55^ for two types of narrow and wide beams. It is noted that the width of cantilever beam for the narrow and wide cases is considered 500 *nm* and 50 *µm*, respectively. Constitutive material properties and geometrical dimensions of the cantilever beams are *G* = 2.5 *µm*, *h* = 1 *µm*, *L* = 300 *µm*, *l* = 100 *nm*, *v* = 0.33, and *E* = 77 *GPa*. Table 1 reveals that the results of the present model are in a good agreement with previous results and the difference between the results is within the range of those of other models^51–55^.

**Table 1:** Comparison the critical voltage of cantilever beams based on different theoretical studies.

	Pull-in voltage (*Volt*)					
	ref. ^51^	ref. ^52^	ref. ^53^	ref. ^54^	ref. ^55^	Present work
Narrow beam	1.23	1.20	1.21	1.21	1.24	1.23
Wide beam	2.27	2.25	2.27	2.16	2.27	2.17

As another verification, the simulation results are compared with the available experimental results. In the following, electrode dimensions and material properties are considered as those reported in ref. ^57^ (*G* = 92 *µm*, *h* = 57 *µm*, *b* = 5 *mm*, *L* = 20 *mm*, and *E* = 155.8 *GPa*). In Fig. 2, relationships between the beam tip deflection and the applied voltage based on Eqs. (13) and (17) is shown. As it can be seen, the electrode deflection gradually increases by applying potential difference. By increasing the voltage until the pull-in instability point, the electrode suddenly adheres to the substrate. Figure 2 reveals that the results of the proposed model agree with experimental data very well. Due to lack of experimental results from nanosystems, the researchers could just compare their models and simulations with available data from systems in the ranges of micro^57^ (Fig. 2) or micro/nano^51–55^ (Table 1).

After validation study, we analyze the system parameters. In the following, a set of parametric studies is presented to investigate effects of main system parameters on the resonance frequency and instability characteristics. The suspended electrode size and constants selected in calculations of this biosensor are *G* = 18 *nm*, *b* = 18 *nm*, *h* = 3.5 *nm*, *L* = 90 *nm*, *l* = 2 *nm*, *τ*_0_ = 0.1 *N/m*, *E*^S^ = 100 *N/m*, *v* = 0.33, and *E* = 176 *GPa*.

When considering a nanosystem, it is worth noting that a cantilever nanobeam may structurally be unstable. It can undergo a primary displacement due to the interactions at ultra-small scales, while no voltage is applied. This effect can induce undesired adhesion in freestanding during the production and operation of such miniature blocks, which should be considered especially when taking small gaps.

**Figure 3:**
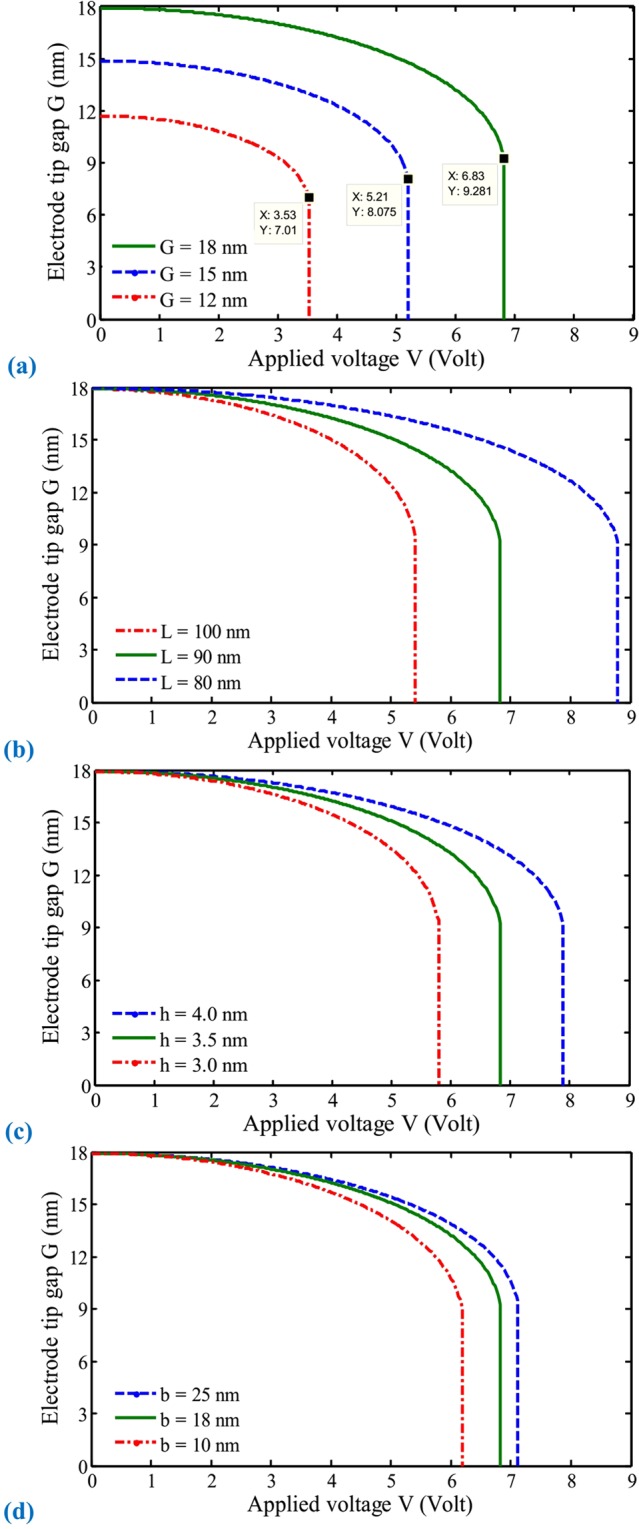
Effects of electrode tip gap (**a**), length (**b**), height (thickness) (**c**), and width (**d**) on the performance of electrically actuated NEMS biosensors.

The relationships between the electrode tip gap and applied voltage at different primary distances between two conductors are displayed in Fig. 3(a). It can be observed that the critical potential difference decreases with decreasing the primary tip gap. It is also found that by considering higher gaps, the relative threshold deflection increases. Moreover, by decreasing the primary gap, the freestanding phenomenon will appear that causes an initial deflection in the movable electrode^58^. This behavior due to molecular forces, which can even result in an undesirable collapse, should be taken into account in the design, analysis, and operation of ultra-small instruments.

Figure 3(b,c) illustrate the variation of the nanoelectrode tip versus the external potential difference at different beam length and height, respectively. It is comprehended that the influence of electrode length and height on the structural stability and response is noteworthy. These figures reveal that the threshold voltage increases with enhancing the beam height, unlike the length. Furthermore, it can be found that by increasing the electrode length, achieving further deformations is possible due to an increase in the operational range of the system.

The variation of the deformable electrode width on the behavior of nanoresonators is presented in Fig. 3(d). The influence of electrode width on the sensitivity and operation of such actuated beams is not as considerable as other geometrical parameters, especially by considering the relatively large width-height ratios. Therefore, to obtain more realistic results, considering the coupled effects of all system parameters is essential. These are basic guidelines in designing mechanical manipulators that should be accounted for NEMS biosensors.

**Figure 4:**
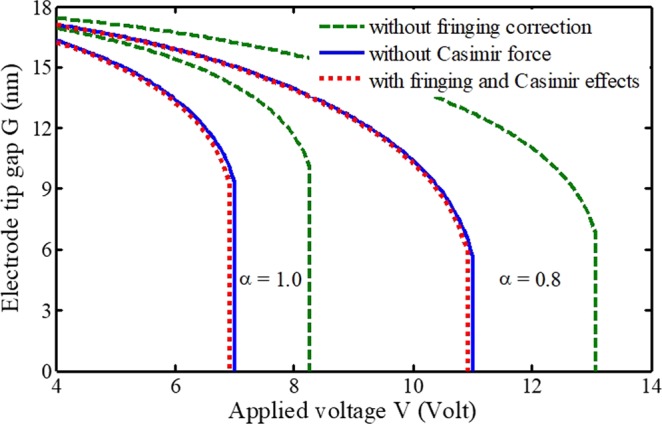
Effects of fringing field correction, Casimir force, and adhering dimension on the performance of NEMS biosensors.

Figure 4 illustrates the relationships between the cantilever tip displacement and the applied voltage, with and without consideration of the fringing field correction as well as the Casimir attraction. It is found that the influence of the fringing field on the stability of electrically actuated nanobeams is noteworthy. The obtained results reveal that taking the fringing field into account makes the beam behave softly and result in declining the critical voltage. Furthermore, depositing larger bioparticles on the substrate results in increasing the differences between the pull-in parameters with and without considering the electric fringing correction.

In addition, the Casimir effect on structural behavior becomes greater by depositing biomaterials. Therefore, the differences between the threshold voltage and maximum deflection with and without modeling the Casimir force is enhanced. Moreover, the effect of the electric fringing correction is more dominant than the Casimir force on the presented biosensor. In general, ignoring the fringing field and the dispersion force lead to an incorrect analysis in micro and nanoscales and the predicted critical voltage will be overestimated. This point is more significant for NEMS biosensors when they are employed to detect different materials in a biosample.

**Figure 5:**
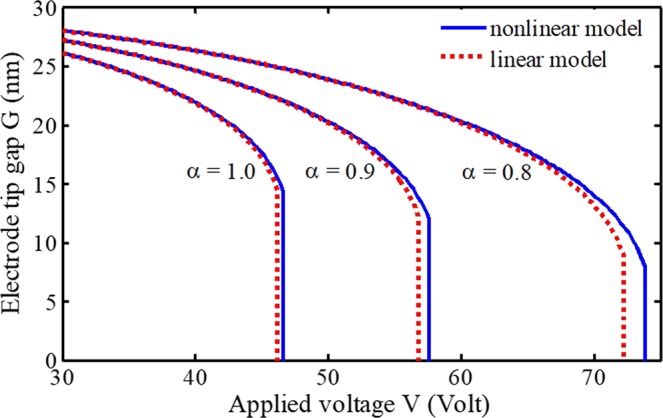
Effects of nonlinear curvature and adhering dimension on the performance of NEMS biosensors.

The dimension effect of the deposited biomaterial on the stability of the indicator is shown in Fig. 5 (*G* = 30 *nm*, *L* = 50 *nm*, *β* = 0). By comparing the curves, it is concluded that the dimension of bioparticles plays an important role in the response of biosensors. In such a biosystem, the actuated area of the substrate is less than the deformable electrode, i.e. *α* or *β* are not zero. As a result, the electrostatic and dispersion forces are smaller than a substrate that is not in a biosample (more explanations about the bioparticle dimension will be given in the following of Table 2). Here, the pull-in phenomenon also happens with a delay, hence, the instability voltage and the maximum deflection become larger.

Figure 5 also illustrates the impact of nonlinear curvature on the predicted voltages is considerable, which makes the cantilever stiffer. As a result, the critical voltage according to the proposed nonlinear model is larger than that predicted by the linear model. It should be mentioned that the results according to the linear theory could be achieved by setting *ξ* = 0 in Eq. (14). Furthermore, it is deduced from the relationship of geometrical nonlinearity that the effect of nonlinearity on the system behavior is more apparent for short beams by accounting considerable gaps. Therefore, as the length decreases and/or the initial gap increases, the differences between the system responses in linear and nonlinear models increase. Therefore, the influence of the geometrical nonlinear deformation becomes more evident. Moreover, Fig. 5 shows that the effect of nonlinear curvature on the critical voltage increases significantly as a biological particle adheres to the substrate. Furthermore, when a larger biomaterial deposits on the fixed conductor, the nonlinearity effect plays a more prominent role on the system performance. Identifying different bioparticles and estimating the dimension from accurate responses in biosensors is not possible without taking the nonlinear curvature into account. These are notable points to describe biosystem behavior, instability conditions, and sensing applications.

As illustrated in these figures, the geometrical properties of both electrodes, as well as their mechanical properties, play key roles when investigating the operating circumstances of NEMS biosensors. To improve the operational range and sensitivity, geometrical parameters also have the ability to adjust the manipulator performance. Furthermore, the effects of mechanical parameters change by varying the scale and size of the structure. Hence, consideration of the geometrical parameters and reflection of their effects in the design and simulation of miniature devices is vital.

**Figure 6:**
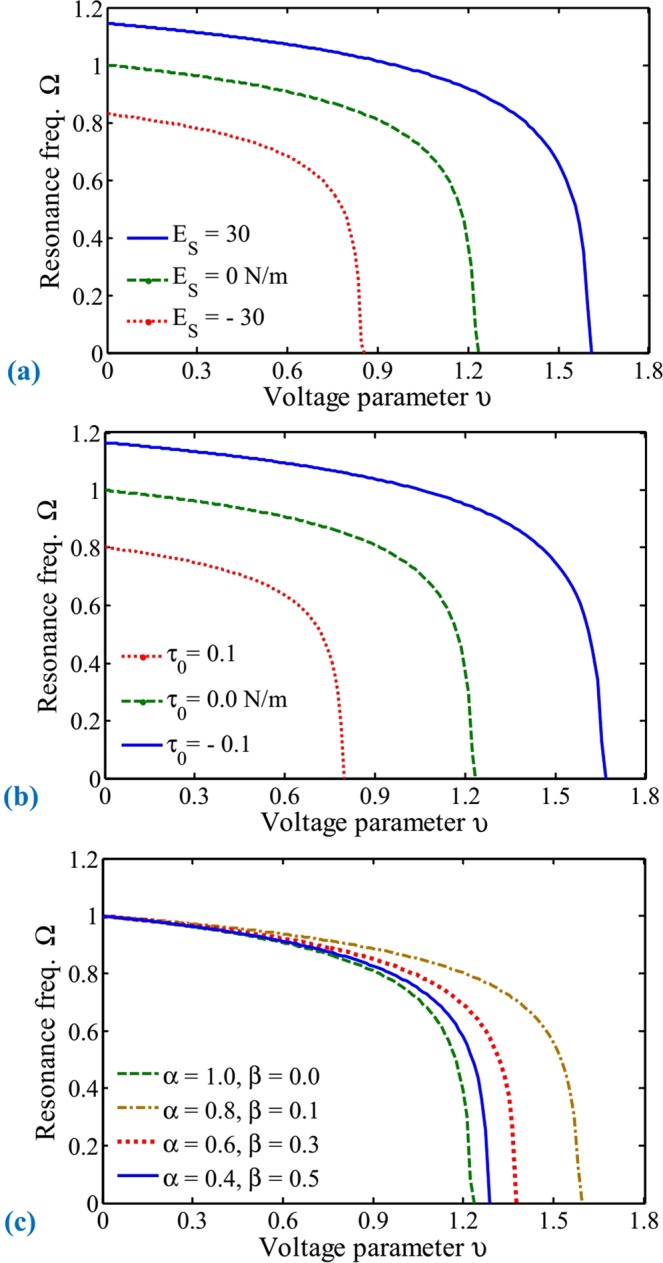
Effects of system parameters on the resonance frequency of electromechanical biosensors for different values of (**a**) surface layer Young’s modulus, (**b**) surface residual stress, and (**c**) adhering position.

Recently, stability and frequency analyses of miniature biosensors were the main objectives of numerous studies^1,2,16,43^. In micro and nanoscales, where the ratio of the external surface of the movable electrode to its volume is relatively considerable, the surface energy effect dominates. This effect is divided into the surface elasticity and residual stress impacts, which perform key roles. To investigate the effect of the surface layer on the resonance frequency and pull-in instability phenomenon of NEMS manipulators, Fig. 6(a,b) are presented. Here, the normalized frequency Ω associated with the non-dimensional voltage *υ* is reported with and without considering any of two surface layer parameters, separately. Frequency normalization is based on consideration of the exact fundamental frequency in the classical macroscale systems, where molecular effects are not significant. The green dashed line is related to the model without consideration of surface layer effects, where the resonance frequency is associated with the classical macro-cantilevers. Moreover, the applied potential difference leading to the electrode collapse is the dynamic voltage.

**Table 2:** Pull-in voltage (*Volt*) and deflection (*nm*) by accounting attracted biological particles at different locations.

*β*	*α*	0.1	0.2	0.3	0.4	0.5	0.6	0.7	0.8
0.10	Voltage	12.17	12.05	11.75	11.25	10.55	9.71	8.77	7.79
	Deflection	7.07	7.12	7.25	7.58	7.94	8.37	8.64	8.75
0.15	Voltage	10.4	10.3	10.1	9.77	9.31	8.71	8.01	7.23
	Deflection	7.25	7.35	7.49	7.68	8.08	8.39	8.66	8.76
0.20	Voltage	9.23	9.19	9.07	8.83	8.47	8.01	7.45	
	Deflection	7.37	7.51	7.71	7.81	7.93	8.31	8.42	
0.25	Voltage	8.57	8.53	8.43	8.23	7.95	7.57	7.09	
	Deflection	7.72	7.74	7.82	7.83	8.08	8.37	8.51	
0.30	Voltage	8.09	8.07	7.97	7.83	7.59	7.25		
	Deflection	7.73	7.91	7.8	7.93	8.12	8.48		
0.35	Voltage	7.75	7.73	7.67	7.51	7.31	7.01		
	Deflection	7.88	7.97	8.13	8.1	8.24	8.62		
0.40	Voltage	7.51	7.49	7.41	7.29	7.09			
	Deflection	8.1	8.16	8.22	8.28	8.36			
0.45	Voltage	7.31	7.29	7.24	7.11	6.95			
	Deflection	8.15	8.21	8.27	8.29	8.71			
0.50	Voltage	7.17	7.15	7.11	6.99				
	Deflection	8.21	8.25	8.53	8.44				
0.55	Voltage	7.07	7.05	6.99	6.89				
	Deflection	8.31	8.31	8.36	8.48				
0.60	Voltage	6.99	6.97	6.93					
	Deflection	8.43	8.44	8.66					
0.65	Voltage	6.93	6.91	6.87					
	Deflection	8.47	8.47	8.66					
0.70	Voltage	6.89	6.87						
	Deflection	8.55	8.54						
0.75	Voltage	6.87	6.85						
	Deflection	8.72	8.71						

The results illustrate that the natural frequency decreases as the electrical force increases and finally approaches zero at the unstable point. It can be seen that neglecting influence of the surface layer leads to obtaining inaccurate results. The obtained results are explained by considering stiffness terms involved in Eq. (15), which contain *λ* or *η* dimensionless parameters induced by the surface layer. It should be noted that both surface layer parameters can be positive or negative depending on the constitutive materials of the movable arm^59,60^. In addition, it has recently been demonstrated that by minimizing the surface stress we are able to further improve the mass sensitivity of clamped-clamped microresonators^43^. Using the relation of the residual surface, it can be understood that the influence of this parameter will increase by increasing the ratio of beam length to the beam thickness. Consequently, this effect is more noteworthy for biological nanodetectors with a slender electrode. The results also demonstrate the importance of considering the mechanical properties in modeling miniature biosensors.

The effects of attracted biological particles on the behaviors of vibrating sensors are shown in Fig. 6(c). The size of adherent particles is considered equal, but they are not the same because they have been attracted to different locations. As mentioned, several points on the substrate surface have been coated with different specific biomaterials as receptors. The term *α* (*β*) indicates the distance between the electrode base (tip) and particle edges (more explanations about *α* and *β* will be presented in the following of Table 2). Note that surface layer effects are neglected in Fig. 6(c) and the green dashed line are figured by considering no adherent particles. It can be found that by investigating the resonance frequency we can recognize the attracted particle on condition that we know its adhering position (*α* and *β*). However, when the substance of the suspended particle in addition to its dimensions are not definite, system identification becomes impossible by applying the frequency analysis. Moreover, it will not be feasible to obtain dimensions of a specific particle by just measuring the system frequency because the adhering position is also effective. The results demonstrate that, in order to identify bioparticles and their size, presenting an applicable and well-organized approach is absolutely necessary.

## Model Applications as a Biosensor

In this research work, the instrumental concept is that system identification is achievable by investigating the delay of instability. The deposition of bioentities restricts the operative actuated area of the unmovable conductor. Consequently, it is required to increase the relative electric potential difference. This fact provides an appropriate and practical method for detecting unspecified entities, adhered to the sensing zone, and their dimension. In practice, measuring just the critical voltage and maximum deflection of a nanosensor is feasible. Hence, it is very important to detect adherent bioparticles by investigating only the mentioned threshold values known as pull-in parameters. We are able to identify attracted particles by assessing measured values at instability conditions if the substrate surface has been coated with different chemicals at specific locations. Determining dimensions of the particle in addition to the identification of its substance is possible via the present biosensor.

The threshold voltage and deflection of the NEMS biosensor are listed in Table 2. These are based on different conditions due to the attraction of a particle in an unspecific location. The principal aim of this table is to detect the attracted particle and estimate its size. It will be performed by investigating both the instability voltage and maximum deflection as measurable input raw data. Furthermore, we can carry out different types of interpolation and extrapolation to identify particles. It should be noted that without using any of the critical voltage or deflection, accurately predicting the cell dimension is unfeasible. It is due to different conditions of adhering biological components.

The non-dimensional terms *α* and *β* indicate the length of effective zones from the base and the tip of the electrode until the particle edges, respectively (see Eqs. (9) and (14)). There are no adherent particles in these zones, so these are effective or actuated zones. Furthermore, the adherent particle blocks the length between *α* and *β*, so it is considered as an unactuated zone. In other words, there exist no electrostatic, fringing and Casimir attractions between the fixed and suspended electrodes in the ineffective zone. Increasing the difference between the values of *α* and *β* means adhering a larger particle on the substrate. It should be mentioned that when no particle adheres to the substrate (*α* = 1 and *β* = 0), the pull-in voltage and deflection are 6.83-*Volt* and 8.77 *nm*, respectively.

It can be realized that by adhering a particle on the electrically actuated fixed conductor, the pull-in voltage increases because the effective actuated length of the substrate conductor decreases. Moreover, the critical electric difference increases by considering a larger adherent particle (by decreasing the total value of *α* and *β*). It means that as the value of *α* or *β* decreases, the threshold voltage always increases. Moreover, the results are more obvious at small values of *β*, but large values of *α*. It is because the beam tip is more sensitive and deformable, so its excitation plays the greatest role in the performance of cantilever detectors. In addition, the beam tip deflection decreases when a particle adheres to the fixed conductor and the pull-in deflection usually decreases by decreasing *α* and *β*. It should be noted that considering target components on the edges of the beam (base or tip) can lead a bit different results, which are not generally accurate. Moreover, by increasing the blocked area, the pull-in phenomenon may not happen in exceptional circumstances as mentioned in ref. ^61^. As another instrumental point, the critical voltage and maximum deflection do not change linearly by adhering superior particles. It demonstrates the significance of implementing quantitative investigations in such nonlinear nanosystems. The results also provide considerable guides to the design and operation of an extensive family of highly-sensitive NEMS biosensors.

Next, some examples are presented to show how input raw data can be employed for system identification. Consider that the measured values of the instability voltage and deflection are 8.0-*Volt* and 8.5 *nm*, respectively. Based on the reported data in Table 1 and by employing interpolation, the values of dimensionless terms *α* and *β* are calculated as 0.66 and 0.17, respectively. Consequently, the length ratio of the attracted particle to the movable electrode is 0.17 (=1 − *α* − *β*). In addition, when different antibodies or receptors have been coated on the substrate with specific distances, we are able to recognize antigens or bioparticles by investigating the pull-in voltage and deflection. It means that by examining coated materials, identification of the particle type (its substance) becomes possible. That is because we know the chemical receptors in that region. Consider that the surface of the substrate has been coated with four different receptors A-D (see Fig. 1(f)). The distances of all four receptors with each other as well as with the ends of the fixed conductor are equal. Inspection of the obtained results reveals the sorbent, in this case, is receptor C. Therefore, by knowing the receptor (antibody), we are able to recognize the adherent analyte (antigen).

As another example, assume that the measured values of voltage and deflection are 7-*Volt* and 8.5 *nm*, respectively. By considering the measured values and the data in Table 1, it is realized that *α* = 0.37 and *β* = 0.52, so the length ratio of the bioparticle is 0.11. In this case, the critical voltage is less than the first example because the tip effective region of the fixed conductor (*β*) is longer and the particle is smaller. We can also identify the attracted particle by understanding its sorbent, i.e. the receptor B.

It is worth noting that determining the adhering location is essential to obtain valid results for detecting biological particles via a comprehensive model. It can also be understood that by measuring either the pull-in voltage or deflection, accurate detection of entities is not feasible. As shown in mentioned two examples, the pull-in deflections were similar, but due to different pull-in voltages, dimensions of bioparticles were not the same. Moreover, adhering locations of particles were not similar because their receptors were different. This conclusion can be obtained by investigating the locations of coated materials as receptors. Finally, it should be noticed that a highly-sensitive device operating as a gage in different conditions must be calibrated to avoid possible measuring errors. It is due to the existence of different sources, such as initial effects of the liquid sample as well as coated materials, which can affect the behaviors of submicron-scale actuated biosensors.

## Conclusions

Several miniature biosensors are recently used to detect living cells and approximate their numbers/dimensions/locations by investigating system responses. In this research, a sensor was introduced to identify biological particles based on applying an electrical excitation. To attract bioparticles, several points of the fixed electrode were coated with different chemicals as receptors. By applying the electric potential and analyzing the pull-in instability characteristics, we are able to recognize adherent entities and their dimension. Here, the governing nonlinear equation was derived by means of Hamilton’s principle. A Galerkin approximation was applied to discretize the nonlinear equation and the SSLM was used to solve them. The accuracy of the system model was validated with available numerical and experimental data. Afterward, the effects of electrode dimensions like thickness, width, length, and initial gap on the performance of biological devices were investigated. It was found that,

- The sensitivity and performance of nanosensors are related to their both mechanical and geometrical properties.
- System identification is not possible by applying a resonance frequency analysis when the substance of the adherent particle, as well as its dimensions, is not definite.
- Increasing the pull-in voltage of NEMS sensors due to the attraction of bioparticles provides an appropriate and practical solution for system identification.
- Determining the dimension of the biological particle in addition to the identification of its substance is possible via the present biosensor by investigating changes in the measured threshold parameters.
- The critical deflection of the electrode in addition to the electric potential difference should be considered to detect different particles due to different conditions of adhering entities.

In practice, measuring just the critical voltage and maximum deflection of a nanosensor is feasible. Hence, it is important to detect adherent particles by investigating only the mentioned threshold values. Investigating the effect of biomaterials in changing the dielectric constant is expected to predict the effective capacitive gap more accurately can be carried out in the future.
